# Supplementary material for: What factors contribute to the Scapular Assistance Test result? A classification and regression tree approach
Source: PLoS One. 2022 Oct 21;17(10):e0276662. doi: 10.1371/journal.pone.0276662 (PMC9586338; doi:10.1371/journal.pone.0276662)
Supplement: Attachment — Submitted filename: Manuscript_CART_PLOSONE from reviewer.docx [file pone.0276662.s002.docx]

**What factors contribute to the Scapular Assistance Test result? A classification and regression tree approach**

Larissa Pechincha Ribeiro, PT, MS^1^, Rodrigo Py Gonçalves Barreto, PT, PhD^1^, Ricardo Augusto Souza Fernandes, Eng, PhD^2^, Paula Rezende Camargo, PT, PhD^1^.

^1^ Laboratory of Analysis and Intervention of the Shoulder Complex, Department of Physical Therapy, Universidade Federal de São Carlos, São Carlos, Brazil.

^2^ Department of Electrical Engineering, Universidade Federal de São Carlos, São Carlos, Brazil.

**Corresponding Author**

Paula Rezende Camargo

Department of Physical Therapy, Universidade Federal de São Carlos, Rodovia Washington Luiz, km 235, São Carlos, São Paulo – Brasil CEP: 13565-905

Email: prcamargo@ufscar.br

Phone number: +55 16 3306 6695

**ABSTRACT**

Alterations in scapular motion are commonly present in individuals with shoulder pain. The Scapular Assistance Test is a maneuver used to investigate whether scapular kinematics may be related with shoulder pain. However, it is not clear the predictors factors associated to positive and negative Scapular Assistance Test. The aim of this study was to determine predictive factors related to the test in individuals with shoulder pain during arm elevation, and to analyze how these predictors interact in a nonlinear manner to discriminate the result of a positive and negative. Eighty-four individuals with shoulder pain were allocated in positive (n=47) and negative (n=37) Scapular Assistance Test. The Scapular Assistance Test result may be influenced by different interactions of contributing factors, where biomechanical variables alone do not respond to the test result and pain catastrophizing may be considered an important predictor for a positive Scapular Assistance Test.

**KEYWORDS:** Dyskinesis, Scapula, Shoulder, Physical therapy, Biopsychosocial.

**HIGHLIGHTS**

- The angular onset of pain during arm elevation was the main predictor for a positive Scapular Assistance Test;

- Biomechanical variables alone do not respond to the Scapular Assistance Test result;

- Pain catastrophizing may be considered a relevant predictor for a positive Scapular Assistance Test;

- Disability of the upper limbs does not seem to contribute to the SAT result.

**INTRODUCTION**

Alterations in scapular motion are commonly described in different conditions of shoulder pain (1–5). The association between scapular motion and clinical presentation may be assessed by symptom modification tests (6–8). The Scapular Assistance Test (SAT) is one of these tests in which the scapula is manually assisted by the examiner into upward rotation and posterior tilt while the patient actively elevates the arm (9). The SAT is considered positive when a reduction of at least two points on the 11-point numerical pain rating scale occurs during assisted elevation as compared to elevation without assistance (9). A positive SAT may suggest that scapular position or motion impairments are likely contributing factors to the painful condition (10).

More recently, a positive SAT was associated with greater pectoralis minor length impairments, showing that other factors not solely based on scapular motion may contribute to a positive SAT (11). Other underlying impairments related to level of disability, pain intensity, scapular muscles strength, and psychological aspects, such as pain catastrophizing, may account and interact for the test result. More studies are needed to support the clinical rationale for the SAT and its relevance. Identifying other factors that may contribute to a positive SAT will enhance the knowledge about the test and possibly guide the clinical decision-making in the management of individuals with shoulder pain.

Therefore, the aim of this study was to determine the predictive factors related to the SAT in individuals with shoulder pain during arm elevation, and to analyze how these predictors interact in a nonlinear manner to discriminate the result of a positive and negative SAT.

**MATERIALS AND METHODS**

**Participants and Study Design**

This is a cross-sectional study. Individuals were included if they had atraumatic self-reported unilateral shoulder pain during flexion of the arm for at least four weeks since first onset and full active arm flexion (~150°) as measured by a clinical inclinometer. Recruitment was performed by advertisements on local websites and printed flyers at the university and in the community. Participants were excluded for any of the following reasons: history of fracture or previous surgery in the upper limbs, presence of neck-related pain determined by the Spurling's and cervical quadrant tests, shoulder pain reproduced by the Upper Limb Tension Test for the median nerve, and recurrent glenohumeral joint dislocations in the last two years.

This study was part of a larger project that was submitted and approved by the Human Research Ethics Committee of Universidade Federal de São Carlos (protocol number 1.394.925). All participants received verbal and written explanation of the study, and those who agreed to participate signed an informed consent.

**Examination procedures**

All participants were examined by one of two physical therapists with at least 3 years of clinical experience related to shoulder disorders. After having the demographics information measured and recorded, SAT, pain intensity at rest, angular onset of pain, scapular dyskinesis, strength of the serratus anterior and lower trapezius, Disabilities of Arm, Shoulder and Hand (DASH) questionnaire and Pain Catastrophizing Scale (PCS) were assessed in all participants. All outcomes were collected at Laboratory of Analysis and Intervention of the Shoulder Complex, Department of Physical Therapy in the same day by the same examiner.

**Scapular Assistance Test**

The maneuver was performed while the individual was actively elevating the arm in the sagittal plane in a standing position. The examiner assisted the scapular upward rotation by pushing upward and laterally on the inferior angle, and the scapular posterior tilt by pulling the superior aspect of the scapula (9). The test was considered positive when individuals reported a decrease in shoulder pain of two or more points on the 11-point numerical pain rating scale during the assisted elevation as compared to the elevation without assistance (9).

**Pain at rest and angular onset of pain**

Presence of pain was assessed by asking the individuals if shoulder pain was present (yes/no) at rest at the moment of data collection. The angular onset of pain during elevation of the arm was measured with a digital inclinometer (AcumarTM Lafayette Instrument Company, Lafayette, IN). Individuals were instructed to assume a relaxed standing position with the arms at their side, and then raise the arm in the sagittal plane until the onset of shoulder pain. The inclinometer was placed distally on the humeral shaft for registration of the angle. Only one trial was performed.

**Scapular dyskinesis**

Scapular dyskinesis was assessed by clinical observation of scapular motion during active, bilateral, and non-weighted elevation of the arm in the sagittal plane. It was considered present when the prominence of the medial scapular border, inferior angle or rapid scapular downward rotation was observed in 3/5 trials of arm elevation (12).

**Muscle strength**

Serratus anterior (SA) and lower trapezius (LT) muscle strength was measured using a handheld dynamometer (Lafayette Instrument Company, Lafayette, IN, USA). These muscles are important scapular movers and stabilizers (Camargo and Neumann, 2019; Neumann and Camargo, 2019). To assess the SA, the participants were in supine with the elbow and arm at 90° (Pires and Camargo, 2018; Ribeiro et al. 2020). The dynamometer was placed on the elbow and the force was applied to the ulna perpendicular to the table. To assess the LT, individuals were in prone with the elbow in extension and arm at 140° of abduction. The dynamometer was placed on the lateral third of the scapula between the acromion and the root of the spine (Michener et al., 2005; Ribeiro et al. 2020).

Three submaximal repetitions of each test were performed for familiarization. Next, three 5-second repetitions of maximal isometric contractions with a 30 second-rest interval between repetitions were performed. Both tests’ positions followed the recommendations of previous studies (10,13,14). A standardized verbal encouragement to develop maximal strength in all contractions was given by the principal investigator in a consistent manner to all participants during the testing procedure. Resistance was manually applied by the examiner who had to keep a constant resistance during the test. The order of assessment of each muscle was randomized.

**Disability of the upper limbs**

The DASH (Disabilities of the Arm, Shoulder, and Hand) questionnaire was used to assess the disability of the upper limbs. It contains 30 questions that are scored on a 5-point rating scale. Final score ranges from 0 to 100. Higher scores indicate the worst possible condition (15).

**Pain catastrophizing**

Pain catastrophizing was measured with the Pain Catastrophizing Scale (PCS). This score comprises 13 questions divided into 3 domains: magnification, helplessness, and rumination. The total score ranges from 0 to 52. Higher scores indicate more catastrophic thoughts (16).

**STATISTICAL ANALYSIS**

Statistical analysis was conducted with Statistical Package for the Social Sciences (SPSS Inc., Chicago, IL) version 25.0. The Kolmogorov-Smirnov test was used to check data distribution. Chi-squared tests were used to compare sex, affected shoulder, pain at rest and scapular dyskinesis. Unpaired Student's t-tests were used to compare height, body mass, serratus anterior muscle strength, DASH and PCS between individuals with positive and negative SAT. Mann-Whitney tests were used to compare age, duration of pain, angular onset of pain and lower trapezius muscle strength between individuals with positive and negative SAT.

Descriptive analyses were used to characterize the sample in relation to the outcome variable of interest the SAT and its selected potential predictors (pain at rest, angular onset of pain, serratus anterior strength, lower trapezius strength, scapular dyskinesis, DASH and PCS). Predictors and characteristics of the participants were compared between those with a positive and negative SAT. The Classification and Regression Tree (CART) analysis was used to determine which factors would predict the occurrence of a positive or negative SAT and possible interactions among them. A CART is a multivariate, nonparametric classification (regression) model that develops a decision tree by successive divisions of the initial set of data, until further divisions are not possible or until pre-established criteria for tree growth are reached (17). For each of these divisions, all possible predictors and their respec­tive cutoff points are considered to establish the predictor that best classifies the individuals into each of the outcome categories (17,18). The order of entry of predictors in the model illustrates hierarchically the strength of association between each predictor and outcome variable, and subsequent divisions identify possible interaction among predictors. The choice of the CART to analyze the data was based on its robust analysis, which captures nonlinear relationships between predic­tors and produces results easily applied in clinical practice (18,19).

For this study, the Weka open-source software was used. The CART was parameterized to present a confidence factor not less than 0.95. The leave-one-out cross-validation process was considered to overcome the possibility of overfitting during the learning (20–22). More specifically, in this cross-validation process, one sample of the set of data is used to validate the CART, while the remaining data are used for training.

To facilitate clinical comprehension of the prediction model produced by the CART, the dependent variable (SAT) was dichotomized as percentiles corresponding to a positive and negative SAT of the sample’s distribution. The area under the receiver-operating characteristic (ROC) curve was calculated to verify the performance of the prediction model. A significance level of 0.05 was established to verify whether the area under the ROC curve was different from 0.5, which would indicate that the model was accurate to predict the outcome categories.

**RESULTS**

A total of 236 individuals were recruited. Figure 1 brings the flow chart of the study. Eighty-four individuals completed the study. The characteristics of the individuals and predictors are presented in Table 1. Individuals with a positive SAT presented angular onset of pain higher in the range of arm flexion as compared to those with a negative SAT (p<0.05, mean difference: 20.35°, 95% CI: 4.70°; 36.01°). Individuals with a positive SAT presented less disability of the upper limbs when compared with individuals with negative SAT (p<0.05, mean difference: 7.58 points, 95% CI: 0.08; 15.08). Individuals with a positive SAT also presented lower score in the PCS than individuals with a negative SAT (p<0.05, mean difference: 7.62 points, 95% CI: 2.56; 12.68). There was not difference for the other outcomes (Table 1).

| **Table 1.** Characteristics of the participants and predictive factors. | | | | |
| --- | --- | --- | --- | --- |
|  | **Positive SAT group (n=47)** | **Negative SAT group (n=37)** | **Mean Difference**  **(95% Confidence Interval)** | ***P*-value** |
| Sex | 17 women  30 men | 21 women  16 men | - | 0.07 |
| Age (years) | 38.49 (SD 14.0) | 37.84 (SD12.1) | -0.65 (-6.43, 5.13) | 0.91 |
| Body mass (kg) | 73.5 (SD 10.22) | 72.5 (SD 14.20) | 2.66 (-6.28, 4.32) | 0.72 |
| Height (m) | 1.72 (SD 0.08) | 1.71 (SD 0.10) | -0.01 (-0.05, 0.03) | 0.61 |
| Affected shoulder | 32 dominant  15 non-dominant | 27 dominant  10 non-dominant | - | 0.64 |
| Duration of pain (months) | 28.51 (SD 48.0) | 26.6 (SD 38.5) | -1.86 (-21.14, 17.41) | 0.76 |
| Pain at rest | 14 yes  33 no | 13 yes 24 no | - | 0.60 |
| Angular onset of pain (degrees) | 134.09 (SD 30.9) | 113.73 (SD 41.2) | -20.35 (-36.01, -4.70) | 0.01* |
| Scapular dyskinesis | 35 yes  12 no | 24 yes  13 no | - | 0.47 |
| Serratus Anterior (Kgf) | 17.82 (SD 6.23) | 16.50 (SD 6.47) | -1.33 (-4.09, 1.41) | 0.33 |
| Lower Trapezius (Kgf) | 9.83 (SD 4.03) | 8.1 (SD 4.46) | -1.66 (-3.52, 0.19) | 0.08 |
| DASH (0 – 100) | 23.57 (SD 15.25) | 31.16 (SD 19.2) | 7.58 (0.08, 15.0) | 0.04* |
| PCS (0 – 52) | 16.51 (SD 11.0) | 24.14 (SD 12.2) | 7.62 (2.49, 12.75) | 0.004* |
| Results are mean and standard deviation (SD). *P<0.05, when statistically significant. Abbreviations: DASH, Disabilities of the Arm, Shoulder, and Hand; PCS, Pain Catastrophizing Scale. Higher scores in the DASH and PCS indicate worse disability and greater catastrophization, respectively. | | | | |

The classification tree selected the angular onset of pain during arm elevation as the first predictor variable of the SAT (Figure 2). Individuals with an angular onset of pain below or equal 65º of arm elevation will present a negative SAT. However, this variable alone did not entirely explain the occurrence of a positive SAT, and scapular dyskinesis was selected as the second predictor. Details about tree divisions, with the respective predictors’ cutoff points and the number and percentage of individuals classified in each subgroup according to the selected predictors are presented in Figure 2.

The predictive model correctly classified 47 of the 47 individuals with positive SAT and 31 of the 37 individuals with negative SAT. Overall, the predictive model obtained an average accuracy of 92.9% and ROC area of 0.96. This demonstrated that the classification of individuals into a positive or negative SAT was not random, nor was it affected by the unbalance between the number of individuals with a positive or negative SAT.

**DISCUSSION**

The findings of this study demonstrated that a positive SAT may be influenced by different interactions of contributing factors. The angular onset of pain during arm elevation was the main predictor of a positive SAT selected by the CART. However, this variable alone did not entirely explain the occurrence of a positive SAT, and other predictors (scapular dyskinesis, pain catastrophizing, pain at rest and strength of the serratus anterior and lower trapezius muscles) were selected by the classification tree. Interestingly, pain catastrophizing was selected multiple times and may be considered an important predictor for a positive SAT. The cutoff points defined by the CART analysis may help clinicians to target factors that may be related to the occurrence of a positive SAT.

Those individuals with an angular onset of pain below 65° of arm elevation had a negative SAT. Due to the small amount of scapular motion at the initial range of arm elevation,(23,24) it is not surprising to have a negative SAT in individuals with onset of pain below 65°. In addition, because most daily and labor activities are usually performed above 65°, other factors may contribute to the perceived pain and functionality of the arm to lead to a positive SAT. Above 65° of arm elevation, the presence of dyskinesis combined with low pain catastrophizing (≤ 6 points at the PCS) contributed to a positive SAT. This observed relationship indicates that the pain of these patients are likely due to altered scapular motion as initially proposed by Kibler (6). Individuals with higher pain catastrophizing (> 6 points at the PCS), pain at rest, and strength of the serratus anterior (< 11.3 Kgf) also had a higher likelihood of presenting a positive SAT.

Pain catastrophizing levels above 30 points indicate individuals susceptible to catastrophize (25). Our study shows that mild levels of pain catastrophizing may moderate the probability of a positive SAT. For example, increased pain catastrophizing levels combined with intermediate values of serratus anterior strength (11.3 to 17.8 Kgf) were associated with a negative SAT, but lower pain catastrophizing levels combined with higher values of serratus anterior strength (> 17.8Kgf) were associated with a positive SAT. The serratus anterior is an important scapular mover (26) and change in pain or range of shoulder elevation during the assistance of the scapula has already been associated as a predictor of better outcome after physical therapy management for shoulder pain (27). Therefore, it is conceivable that increasing serratus anterior strength and reducing pain catastrophizing levels can lead to a better prognosis for individuals with shoulder pain as these factors are associated with a positive SAT. For individuals with pain during movement and scapular dyskinesis, the mediating effect of pain catastrophizing seems even clearer as higher levels of pain catastrophizing were associated with a negative SAT.

When scapular dyskinesis is not present, strength of the lower trapezius should be assessed along with pain catastrophizing and strength of the serratus anterior. It is worthy to note that pain at rest was only selected when scapular dyskinesis was present, and that strength of the lower trapezius was only selected in the absence of scapular dyskinesis. Based on the muscle position, LT is most aligned to execute scapular upward rotation during arm abduction when compared to flexion (28). However, SAT was performed during the arm flexion in the current study. Future research may apply the SAT during abduction to investigate the lower trapezius muscle strength deficits. Kibler (6) suggested that a positive SAT was related to a deficit in muscle activation or strength of the scapular muscles. However, recent studies (10,11) did not identify differences in the serratus anterior and lower trapezius muscle strength between those with a positive and negative SAT. In the current study, muscle strength of the serratus anterior and lower trapezius alone could not explain the occurrence of a positive SAT. The inclusion of pain catastrophizing by the CART suggests that the SAT may be influenced not only by biomechanical variables but also by psychological factors.

Negative psychological factors have been reported in individuals with shoulder pain. These factors include emotional aspects such as anxiety and depression,(29) kinesiophobia,(30) fear and avoidance beliefs (31) and pain catastrophization.(32). Recently, individuals with worse psychological profile were shown to present worse function and higher shoulder pain intensity.(33) In the present study, individuals with negative SAT presented higher pain catastrophizing thoughts when compared to individuals with a positive SAT. Clinicians should pay attention to individuals with a negative SAT as they may benefit of an approach that targets psychological factors in addition to therapeutic exercises. Further research should investigate the influence of other psychological factors, such as kinesiophobia, self-efficacy, optimism and resilience that may also predict the SAT result.

From the resulting tree, the cutoff points adjusted during the training stage are accurate since the classification leaves (in blue) mostly separate the two classes accordingly. Analyzing the tree, two leaves presented a misclassification of 6 individuals: (i) 5 individuals in the leaf that used the PCS with a cutoff point above 8 points; and (ii) 1 individual in the leaf that used the PCS with a cutoff point above 15 points. Both leaves are predominantly composed of individuals with positive SAT meaning that these leaves are better suited to classify individuals with positive SAT.

In summary, the resulting tree has 7 levels that combine the following variables: angular onset of pain, presence of scapular dyskinesis, pain catastrophizing, serratus anterior and lower trapezius muscle strength. Considering the results, it can be said that these variables are the most relevant to distinguish between individuals with positive and negative SAT. It is important to note that patients with negative SAT presents better function, but also higher levels of pain catastrophizing when compared to positive SAT. Clinicians should pay attention that “good outcome” is not enough when the patient also presents poor psychosocial aspects. The DASH was not a relevant variable in the tree, even with a significant difference between groups.

This study has some limitations. Although a 2-point decrease in pain intensity was considered for a positive SAT, a register before and after the SAT was not registered. The SAT was performed during flexion of the arm and our findings cannot be extrapolated to other planes of arm elevation. Finally, other psychological factors could have been evaluated to a better understanding of the contribution to SAT results.

**CONCLUSION**

The SAT result was influenced by different interactions of contributing factors, such as angular onset of pain during arm elevation, scapular dyskinesis, pain catastrophizing, pain at rest and serratus anterior and lower trapezius muscle strength. This indicates that the SAT result may be explained not only by biomechanical variables, but also by psychological factors. Disability of the upper limbs does not seem to contribute to the SAT result.

**ACKNOWLEDGEMENT**

We would like to thank Coordenação de Aperfeiçoamento de Pessoal de Nível Superior and Conselho Nacional de Desenvolvimento Científico e Tecnológico (144436/2019-1).

**REFERENCES**

1.	Kijima T, Matsuki K, Ochiai N, Yamaguchi T, Sasaki Y, Hashimoto E, et al. In vivo 3-dimensional analysis of scapular and glenohumeral kinematics: Comparison of symptomatic or asymptomatic shoulders with rotator cuff tears and healthy shoulders. J Shoulder Elb Surg [Internet]. 2015;24(11):1817–26. Available from: http://dx.doi.org/10.1016/j.jse.2015.06.003

2.	Lawrence RL, Braman JP, Laprade RF, Ludewig PM. Comparison of 3-Dimensional Shoulder Complex Kinematics in Individuals With and Without Shoulder Pain, Part 1: Sternoclavicular, Acromioclavicular, and Scapulothoracic Joints. J Orthop Sport Phys Ther [Internet]. 2014;44(9):636-A8. Available from: http://www.jospt.org/doi/10.2519/jospt.2014.5339

3.	Ogston JB, Ludewig PM. Differences in 3-dimensional shoulder kinematics between persons with multidirectional instability and asymptomatic controls. Am J Sports Med. 2007;35(8):1361–70.

4.	Ludewig PM, Cook TM. Alterations in shoulder kinematics and associated muscle activity in people with symptoms of shoulder impingement. Phys Ther [Internet]. 2000;80(3):276–91. Available from: http://www.ncbi.nlm.nih.gov/pubmed/10696154

5.	Kim D, Lee B, Yeom J, Cha J, Han J. Three-dimensional in vivo comparative analysis of the kinematics of normal shoulders and shoulders with massive rotator cuff tears with successful conservative treatment. Clin Biomech [Internet]. 2020;75(December 2019):104990. Available from: https://doi.org/10.1016/j.clinbiomech.2020.104990

6.	Kibler W Ben. The Role of the Scapula in Shoulder Function. Am J Sports Med. 1998;26(2):325–37.

7.	Tate AR, McClure P, Kareha S, Irwin D. Effect of the Scapula Reposition Test on Shoulder Impingement Symptoms and Elevation Strength in Overhead Athletes. J Orthop Sport Phys Ther [Internet]. 2008;38(1):4–11. Available from: doi: 10.2519/jospt.2008.2616.

8.	Lewis JS, McCreesh K, Barratt E, Hegedus EJ, Sim J. Inter-rater reliability of the Shoulder Symptom Modification Procedure in people with shoulder pain. BMJ Open Sport Exerc Med [Internet]. 2016;2(1):e000181. Available from: http://bmjopensem.bmj.com/lookup/doi/10.1136/bmjsem-2016-000181

9.	Rabin A, Irrgang JJ, Fitzgerald GK, Eubanks A. The Intertester Reliability of the Scapular Assistance Test. J Orthop Sport Phys Ther [Internet]. 2006;36(9):653–60. Available from: http://www.jospt.org/doi/10.2519/jospt.2006.2234

10.	Ribeiro LP, Barreto RPG, Pereira ND, Camargo PR. Comparison of scapular kinematics and muscle strength between those with a positive and a negative Scapular Assistance Test. Clin Biomech [Internet]. 2020;73:166–71. Available from: https://doi.org/10.1016/j.clinbiomech.2019.12.030

11.	Grimes JK, Puentedura EJ, Cheng MS, Seitz AL. Scapular movement impairments in individuals with subacromial pain syndrome based on scapular assistance test and scapula reposition test outcomes. Musculoskelet Sci Pract [Internet]. 2020;49(June):102214. Available from: https://doi.org/10.1016/j.msksp.2020.102214

12.	McClure PW, Tate AR, Kareha S, Irwin D, Zlupko E. A clinical method for identifying scapular dyskinesis, part 1: reliability. J Athl Train [Internet]. 2009;44(2):160–4. Available from: doi: 10.4085/1062-6050-44.2.160

13.	Michener L a, Boardman ND, Pidcoe PE, Frith AM. Scapular muscle tests in subjects with shoulder pain and functional loss: reliability and construct validity. Phys Ther [Internet]. 2005;85(11):1128–38. Available from: https://pubmed.ncbi.nlm.nih.gov/16253043/

14.	Pires ED, Camargo PR. Analysis of the kinetic chain in asymptomatic individuals with and without scapular dyskinesis. Clin Biomech [Internet]. 2018;54(February):8–15. Available from: https://doi.org/10.1016/j.clinbiomech.2018.02.017

15.	Orfale AG, Araújo PMP, Ferraz MB, Natour J. Translation into Brazilian Portuguese, cultural adaptation and evaluation of the reliability of the Disabilities of the Arm, Shoulder and Hand Questionnaire. Brazilian J Med Biol Res [Internet]. 2005 Feb;38(2):293–302. Available from: https://doi.org/10.1590/S0100-879X2005000200018

16.	Sehn F, Chachamovich E, Vidor LP, Dall-Agnol L, Custódio de Souza IC, Torres ILS, et al. Cross-Cultural Adaptation and Validation of the Brazilian Portuguese Version of the Pain Catastrophizing Scale. Pain Med [Internet]. 2012;13(11):1425–35. Available from: doi/10.1111/j.1526-4637.2012.01492.x

17.	Gordon AD, Breiman L, Friedman JH, Olshen RA, Stone CJ. Classification and Regression Trees. Biometrics. 1984;

18.	Falconer JA, Naughton BJ, Dunlop DD, Roth EJ, Strasser DC, Sinacore JM. Predicting stroke inpatient rehabilitation outcome using a classification tree approach. Arch Phys Med Rehabil. 1994;

19.	Lemon SC, Roy J, Clark MA, Friedmann PD, Rakowski W. Classification and Regression Tree Analysis in Public Health: Methodological Review and Comparison with Logistic Regression. Annals of Behavioral Medicine. 2003. p. 172–81.

20.	Pasini A. Artificial neural networks for small dataset analysis. J Thorac Dis. 2015;7:953–60.

21.	Rushing C, Bulusu A, Hurwitz HI, Nixon AB, Pang H. A leave-one-out cross-validation SAS macro for the identification of markers associated with survival. Comput Biol Med. 2015;57:123–9.

22.	Jayawardana K, Schramm SJ, Tembe V, Mueller S, Thompson JF, Scolyer RA, et al. Identification, review, and systematic cross-validation of microRNA prognostic signatures in metastatic melanoma. J Invest Dermatol. 2016;136:245–54.

23.	Inman VT, Saunders JB, Abbott LC. Observations of the function of the shoulder joint. Clin Orthop Relat Res. 1944;(330):3–12.

24.	Lee B, Kim D, Jang Y, Jin H. Three-dimensional in vivo scapular kinematics and scapulohumeral rhythm: a comparison between active and passive motion. J Shoulder Elb Surg [Internet]. 2020;29(1):185–94. Available from: https://doi.org/10.1016/j.jse.2019.05.036

25.	Wijma AJ, van Wilgen CP, Meeus M, Nijs J. Clinical biopsychosocial physiotherapy assessment of patients with chronic pain: The first step in pain neuroscience education. Physiother Theory Pract. 2016;32(5):368–84.

26.	Neumann DA, Camargo PR. Kinesiologic considerations for targeting activation of scapulothoracic muscles: part 1: serratus anterior. Brazilian J Phys Ther [Internet]. 2019;(xx):1–8. Available from: https://doi.org/10.1016/j.bjpt.2019.01.008

27.	Chester R, Jerosch-Herold C, Lewis J, Shepstone L. Psychological factors are associated with the outcome of physiotherapy for people with shoulder pain: A multicentre longitudinal cohort study. Br J Sports Med. 2018;52(4):269–75.

28.	Camargo PR, Neumann DA. Kinesiologic considerations for targeting activation of scapulothoracic muscles – part 2: trapezius. Brazilian J Phys Ther [Internet]. 2019;(xx):1–9. Available from: https://doi.org/10.1016/j.bjpt.2019.01.011

29.	Cho CH, Jung SW, Park JY, Song KS, Yu KI. Is shoulder pain for three months or longer correlated with depression, anxiety, and sleep disturbance? J Shoulder Elb Surg [Internet]. 2013;22(2):222–8. Available from: http://dx.doi.org/10.1016/j.jse.2012.04.001

30.	Feleus A, Van Dalen T, Bierma-Zeinstra SMA, Bernsen RMD, Verhaar JAN, Koes BW, et al. Kinesiophobia in patients with non-traumatic arm, neck and shoulder complaints: A prospective cohort study in general practice. BMC Musculoskelet Disord. 2007;8:1–11.

31.	Kromer TO, Sieben JM, de Bie RA, Bastiaenen CHG. Influence of Fear-Avoidance Beliefs on Disability in Patients With Subacromial Shoulder Pain in Primary Care: A Secondary Analysis. Phys Ther [Internet]. 2014;94(12):1775–84. Available from: https://academic.oup.com/ptj/article-lookup/doi/10.2522/ptj.20130587

32.	George SZ, Wallace MR, Wright TW, Moser MW, Iii HG, Sack BK, et al. Evidence for a biopsychosocial influence on shoulder pain: Pain catastrophizing and catechol-O-methyltransferase (COMT) diplotype predict clinical pain ratings. Pain. 2008;136:53–61.

33.	Kamonseki DH, Pott-Junior H, Haik MN, Almeida LA de, Camargo PR. Pain-related fear phenotypes are associated with function of the upper limbs in individuals with shoulder pain. Musculoskelet Sci Pract [Internet]. 2021 Oct [cited 2021 Jul 1];55:102416. Available from: https://pubmed.ncbi.nlm.nih.gov/34175789/
