# Supplementary material for: What factors contribute to the Scapular Assistance Test result? A classification and regression tree approach
Source: PLoS One. 2022 Oct 21;17(10):e0276662. doi: 10.1371/journal.pone.0276662 (PMC9586338; doi:10.1371/journal.pone.0276662)
Supplement: Attachment — Submitted filename: Reviewer.docx [file pone.0276662.s003.docx]

Review

**What factors contribute to the Scapular Assistance Test result? A classification and regression tree approach**

**ABSTRACT**

1. Describe the group of factors used in the analysis such as biomechanics, psychological ... what else?
2. The results about the angular of onset which was the main predictor should be addressed in the abstract.

**INTRODUCTION**

1. The first sentence of second paragraph should be rewritten. The pectoralis minor length is not other factors but it should be considered as the underlying factors influencing the abnormal motion of scapular.
2. The underlying impairments reported to contribute to scapular movements and then could influence SAT should be listed. The factors might also should be introduce in categories as the authors addressed in the discussion.

**Methods**

1. 150 degrees should not be considered as full active elevation
2. Please describe the test procedure of Scapular Assistance Test clearly. Did you have the participants perform movement before assisant was provide. How many times the participants have to perform elevation? When and how you evaluate the pain? Etc
3. Did you also assess the pain intensity during SAT. Please report the change of pain intensity in both positive and negative SAT groups.
4. The Scapular dyskinesis test was tested in which plane?
5. For muscle strength test, Describe the direction of movement that used for producing isometric contraction of SA and LT.

**Results**

1. Table 1 should present mean±SD and number (%)

**Discussion**

1. What is kgf?
2. If the scapular dyskinesis is not present, how the assisting force and direction applied during the SAT?
3. So the subject with high level of pain catastrophizing tend to have negative or positive results of SAT?
4. Please discuss more about clinical implication of the findings. Which factors or outcomes should be considered in people with positive SAT?

Overall

Please recheck the grammar and format of inserted references.
